# Tetra­kis{4-[(2*H*-tetra­zol-5-yl)meth­yl]morpholin-4-ium} dodeca­tungstosilicate hexa­hydrate

**DOI:** 10.1107/S1600536811001772

**Published:** 2011-01-29

**Authors:** Mohammad Yousefi, Hossein Eshtiagh-Hosseini, Masoud Mirzaei, Ahmad Gholizadeh, Mohsen Nikpour

**Affiliations:** a Department of Chemistry, Islamic Azad University, Shahr-e Rey Branch, Tehran, Iran; b Department of Chemistry, School of Sciences, Ferdowsi University of Mashhad, Mashhad 917791436, Iran; c Department of Chemistry, Islamic Azad University, North Tehran Branch, Tehran, Iran; d Department of Chemistry, School of Sciences, Islamic Azad University, Ahvaz Branch, Ahvaz 61349-68875, Iran

## Abstract

The crystal structure of the title compound, (C_6_H_12_N_5_O)_4_[W_12_(SiO_4_)O_36_]·6H_2_O, consists of an α-Keggin-type [W_12_(SiO_4_)O_36_]^4−^ polyoxidoanion, four [(2*H*-tetra­zol-5-yl)meth­yl]morpholinium cations and six uncoordinated water mol­ecules. In the cations, the morpholine rings display chair conformations. Extensive N—H⋯O, N—H⋯N, O—H⋯O and O—H⋯N hydrogen bonds are present in the crystal structure.

## Related literature

For applications of polyoxidometalate-based hybrid materials as catalysts, non-linear optical materials and anti-viral drugs, see: Coronado & Gómez-García (1998 ▶). For inorganic–organic hybrid materials based upon polyoxidometalates bearing organic bases, see: Alizadeh *et al.* (2006 ▶, 2008 ▶); Nikpour *et al.* (2009 ▶).
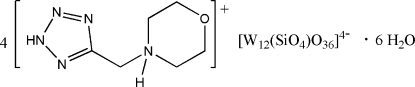


## Experimental

### 

#### Crystal data

(C_6_H_12_N_5_O)_4_[W_12_(SiO_4_)O_36_]·6H_2_O
*M* *_r_* = 3663.21
Triclinic, 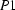

*a* = 12.4512 (5) Å
*b* = 13.1805 (5) Å
*c* = 13.4065 (6) Å
α = 93.856 (1)°
β = 116.331 (1)°
γ = 116.450 (1)°
*V* = 1666.58 (12) Å^3^
*Z* = 1
Mo *K*α radiation
μ = 20.75 mm^−1^
*T* = 100 K
0.32 × 0.16 × 0.11 mm

#### Data collection

Bruker SMART APEXII CCD area-detector diffractometer
Absorption correction: multi-scan (*SADABS*; Sheldrick, 1996 ▶) *T*_min_ = 0.024, *T*_max_ = 0.164
33857 measured reflections
17355 independent reflections
16382 reflections with *I* > 2σ(*I*)
*R*_int_ = 0.030

#### Refinement

*R*[*F* ^2^ > 2σ(*F* ^2^)] = 0.031
*wR*(*F* ^2^) = 0.062
*S* = 1.03
17355 reflections
965 parameters
15 restraints
H-atom parameters constrained
Δρ_max_ = 2.91 e Å^−3^
Δρ_min_ = −3.38 e Å^−3^
Absolute structure: Flack (1983 ▶), 8497 Friedel pairs
Flack parameter: 0.377 (9)

### 

Data collection: *APEX2* (Bruker, 2005 ▶); cell refinement: *SAINT* (Bruker, 2005 ▶); data reduction: *SAINT*; program(s) used to solve structure: *SHELXTL* (Sheldrick, 2008 ▶); program(s) used to refine structure: *SHELXTL*; molecular graphics: *SHELXTL*; software used to prepare material for publication: *SHELXTL*.

## Supplementary Material

Crystal structure: contains datablocks I, global. DOI: 10.1107/S1600536811001772/xu5096sup1.cif

Structure factors: contains datablocks I. DOI: 10.1107/S1600536811001772/xu5096Isup2.hkl

Additional supplementary materials:  crystallographic information; 3D view; checkCIF report

## Figures and Tables

**Table 1:** Hydrogen-bond geometry (Å, °)

*D*—H⋯*A*	*D*—H	H⋯*A*	*D*⋯*A*	*D*—H⋯*A*
N3*A*—H3*NA*⋯O1*W*^i^	0.88	2.01	2.831 (16)	155
N3*B*—H3*NB*⋯O2*W*^ii^	0.88	1.98	2.812 (17)	157
N3*C*—H3*NC*⋯O4*W*^iii^	0.88	1.83	2.69 (2)	167
N3*D*—H3*ND*⋯O3*W*	0.88	1.84	2.69 (2)	163
N5*A*—H5*NA*⋯O2*W*	0.90	1.93	2.797 (19)	162
N5*B*—H5*NB*⋯O1*W*	0.90	1.90	2.740 (18)	154
N5*C*—H5*NC*⋯N1*D*^ii^	0.90	1.99	2.89 (2)	172
N5*D*—H5*ND*⋯N1*C*^i^	0.90	2.07	2.946 (18)	164
O1*W*—H1*WA*⋯O5*W*^iv^	0.85	1.91	2.691 (13)	153
O1*W*—H1*WB*⋯O19	0.85	1.95	2.779 (12)	167
O2*W*—H2*WA*⋯O6*W*	0.85	1.80	2.601 (14)	157
O2*W*—H2*WB*⋯O12^v^	0.85	2.10	2.924 (14)	162
O3*W*—H3*WA*⋯O5*W*^i^	0.85	2.27	2.877 (16)	128
O3*W*—H3*WB*⋯O35^i^	0.85	2.12	2.937 (12)	160
O4*W*—H4*WA*⋯O2^vi^	0.85	2.28	2.862 (14)	126
O4*W*—H4*WB*⋯N4*D*	0.85	2.50	2.89 (2)	109
O5*W*—H5*WA*⋯N2*D*^ii^	0.85	2.31	3.155 (18)	175
O5*W*—H5*WB*⋯O26	0.85	2.17	2.944 (14)	151
O5*W*—H5*WB*⋯O28	0.85	2.45	3.118 (14)	136
O6*W*—H6*WA*⋯O17^vi^	0.85	2.17	2.923 (15)	148
O6*W*—H6*WA*⋯O26^v^	0.85	2.28	2.858 (17)	125
O6*W*—H6*WB*⋯N2*C*^vii^	0.85	2.07	2.911 (17)	172

Symmetry codes: (i) 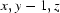; (ii) 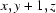; (iii) 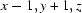; (iv) 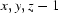; (v) 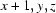; (vi) 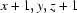; (vii) 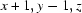.

**Fig. 1.**
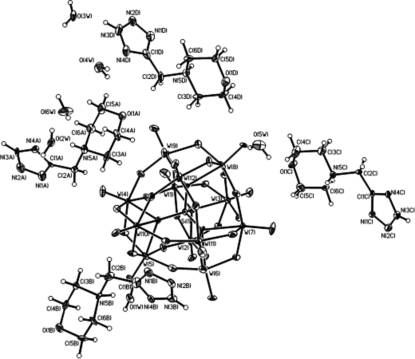
Independent part of unit cell [C6H12N5O]4[W12(SiO4)O36].6H2O, with thermal ellipsoids drawn at 50% probability level.

**Fig. 2.**
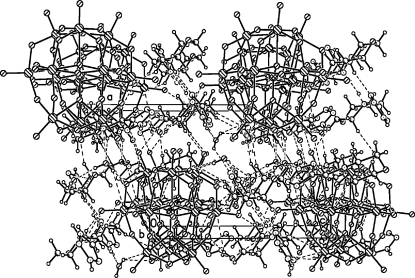
The fragment of crystal packing (projection along b-crystal axis). Complicated three-dimensional-hydrogen bonded network is formed.

**Fig. 3.**
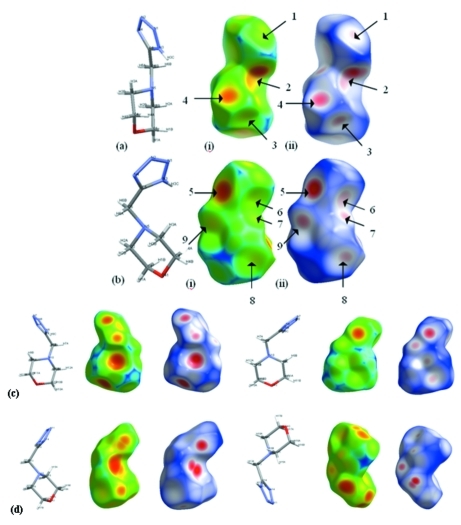
The Hirshfeld surface of [C6H12N5O](I), (a) & (b), [C6H12N5O](II) (c) and [C6H12N5O] (III) (d), mapped withde (i) and the dnorm (ii) property in two orientation. The molecules beside thesurfaces have been added for clarity.

## supplementary crystallographic information

### Comment

In recent years the literature has contained a rapidly growing number of reports concerning the use of polyoxometalate-based hybrid materials as catalysts, non-linear optical materials and anti-viral drugs (Coronado & Gómez-García, 1998). Continuing our previous studies on synthesis and X-ray structure determination of someKeggin type heteropolyoxometalate-based organic inorganic hybrid compounds such as [C_5_H_10_NO_2_][Mo_12_(PO_4_)O_36_].4.5H_2_O (Alizadeh *et al.*, 2006), [L—C_5_H_10_NO_2_]_4_[W_12_(SiO_4_)O_36_].4H_2_O (Alizadeh *et al.*, 2008), and [C_6_H_12_N_5_O]_3_[Mo_12_(PO_4_)O_36_].6H_2_O (Nikpour *et al.*, 2009), herein, we wish to report on the synthesis and crystal structure of a new inorganic-organic hybrid material based upon [W_12_(SiO_4_)O_36_]^4-^ hetropolyoxoanion. The title hybrid compound consists of one [W_12_(SiO_4_)O_36_]^4-^ polyoxoanion, four crystallographically independent [((1*H*-tetrazol-5-yl)methyl) morpholine] cations and six crystallization water of molecules (Fig. 1). The inorganic anion shows a classical α-Keggin structure with four different types of O atoms. This includes 12 terminal O atoms, 4 O atoms that are bonded to Si and W atoms, 12 corner-sharing and 12 e dge-sharing O atoms that both are part of distorted WO_6_ octahedra. The central SiO_4_ tetrahedron is slightly distorted and is surrounded by 12 distorted WO_6_ octahedra. The Si—O bond lengths range from 1.606 (7) to 1.652 (7) Å, and the O—Si—O angles are in the range of 108.8 (4)–111.4 (4)°. All four organic cations show slight differences in bond lengths, angles and torsion angles. They are involved in an extensive hydrogen bonding. Several N—H···O (N···O distances are in the range of 2.69 (2) to 2.831 (16) Å), N—H···N (N···N distances are in the range of 2.89 (2) to 2.946 (18) Å), O—H···O (O···O distances are in the range from 2.601 (14) to 2.944 (14) Å), and O—H···N (O···N distances are in the range from 2.89 (2) to 3.155 (18) Å) hydrogen bonds between the organic cations, inorganic anions and crystallization water of molecules lead to the construction of a three-dimensional– supramolecular framework (Fig. 2). Moreover, six uncoordinated water molecules increase the number of hydrogen bonds in the crystalline network and lead to the formation of (H_2_O)_∞_ clusters throughout the crystalline network.

In the title hybrid,owing to the presence of organic molecules, water molecule and the molecular nature of the compounds, a large numbers of van der Waals interactions, in particular hydrogen bonding interactions have been observed. three-dimensional-isosurface pictures of Hirshfeld surfaces, d_norm_ and d_e_maps, for organic moieties have been shown in the Fig. 3.

In Fig.3(*a*)(i & ii) the region labelled 1 is an intermolecular contact between nitrogenatom of 1*H*-tetrazole ring of organic moiety (I) and oxygen atom of water molecule. The hydrogen bonds from morpholine ring oforganic component (I) and oxygen atoms of polyoxomolybdate can be seen in red-yellow regions labelled 3 & 4. In Fig. 3(*b*)(i)/(ii), the red concave areas denoted 5 reveal thestrong interaction from nitrogen atom of tetrazole ring and water molecule incrystal network; the regions labelled 6–9 show weak interactions of C—H···O(water/polyoxomolybdate). For organic moieties 2 & 3, same interactions have been observe (See Fig. 3(*c*) and Fig. 3(*d*)) but their contributions in intermolecular interactions are different.

### Experimental

A solution of ((1*H*-tetrazole-5-yl)methyl)morpholine (0.17 g, 1.0 mmol) in 30 ml of water was added with vigorous stirring, to a solution of α-H_3_[W_12_(SiO_4_)O_36_].21(H_2_O) (0.48 g, 0.27 mmol) in 25 ml of water for synthesis of the title compound. The colorless precipitate was formed after several hours. The solid was filtered, washed with DMF. The precipitate was redissolved in acetonitrile and the solution was cooled to ambient temperature, colorless prism crystals were obtained.

### Refinement

H atoms bonded to O and N atoms were found in a difference Fourier map, and refined with distance restraints of O—H = 0.85 and N—H = 0.88–0.90 Å. Methylene H atoms were placed in calculated positions with C—H = 0.99 Å and refined in riding model with *U*_iso_(H) = 1.2*U*_eq_(C).

### Crystal data

**Table:**

(C_6_H_12_N_5_O)_4_[W_12_(SiO_4_)O_36_]·6H_2_O	*Z* = 1
*M**_r_* = 3663.21	*F*(000) = 1646
Triclinic, *P*1	*D*_x_ = 3.650 Mg m^−^^3^
Hall symbol: P 1	Mo *K*α radiation, λ = 0.71073 Å
*a* = 12.4512 (5) Å	Cell parameters from 7382 reflections
*b* = 13.1805 (5) Å	θ = 2.3–34.9°
*c* = 13.4065 (6) Å	µ = 20.75 mm^−^^1^
α = 93.856 (1)°	*T* = 100 K
β = 116.331 (1)°	Prism, colourless
γ = 116.450 (1)°	0.32 × 0.16 × 0.11 mm
*V* = 1666.58 (12) Å^3^	

### Data collection

**Table:**

Bruker SMART APEXII CCD area-detector diffractometer	17355 independent reflections
Radiation source: fine-focus sealed tube	16382 reflections with *I* > 2σ(*I*)
graphite	*R*_int_ = 0.030
ω scans	θ_max_ = 29.0°, θ_min_ = 1.8°
Absorption correction: multi-scan (*SADABS*; Sheldrick, 1996)	*h* = −16→16
*T*_min_ = 0.024, *T*_max_ = 0.164	*k* = −17→17
33857 measured reflections	*l* = −18→18

### Refinement

**Table:**

Refinement on *F*^2^	Secondary atom site location: difference Fourier map
Least-squares matrix: full	Hydrogen site location: mixed
*R*[*F*^2^ > 2σ(*F*^2^)] = 0.031	H-atom parameters constrained
*wR*(*F*^2^) = 0.062	*w* = 1/[σ^2^(*F*_o_^2^) + (0.0033*P*)^2^ + 14.9946*P*] where *P* = (*F*_o_^2^ + 2*F*_c_^2^)/3
*S* = 1.03	(Δ/σ)_max_ = 0.003
17355 reflections	Δρ_max_ = 2.91 e Å^−^^3^
965 parameters	Δρ_min_ = −3.38 e Å^−^^3^
15 restraints	Absolute structure: Flack (1983), 8497 Friedel pairs
Primary atom site location: structure-invariant direct methods	Flack parameter: 0.377 (9)

### Special details

**Table:**

Experimental. ^1^HNMR: ( D_2_O) d, 3.40 (t,4H, (CH_2_)_2_N),3.93 (t, 4H, (CH_2_)_2_O), 4.67 (s, 2H, CH_2_-(N(CH_2_)_2_)); ms: m/z , 169. Anal. calcd. for C_24_H_60_N_20_O_50_SiW_12_:C,7.87; H, 1.64; N, 7.64. Found: C, 8.24; H, 1.54; N, 7.97%.
Geometry. All e.s.d.'s (except the e.s.d. in the dihedral angle between two l.s. planes) are estimated using the full covariance matrix. The cell e.s.d.'s are taken into account individually in the estimation of e.s.d.'s in distances, angles and torsion angles; correlations between e.s.d.'s in cell parameters are only used when they are defined by crystal symmetry. An approximate (isotropic) treatment of cell e.s.d.'s is used for estimating e.s.d.'s involving l.s. planes.
Refinement. Refinement of *F*^2^ against ALL reflections. The weighted *R*-factor *wR* and goodness of fit *S* are based on *F*^2^, conventional *R*-factors *R* are based on *F*, with *F* set to zero for negative *F*^2^. The threshold expression of *F*^2^ > σ(*F*^2^) is used only for calculating *R*-factors(gt) *etc*. and is not relevant to the choice of reflections for refinement. *R*-factors based on *F*^2^ are statistically about twice as large as those based on *F*, and *R*- factors based on ALL data will be even larger.

### Fractional atomic coordinates and isotropic or equivalent isotropic displacement parameters (Å^2^)

**Table:**

	*x*	*y*	*z*	*U*_iso_*/*U*_eq_	
W1	−0.15077 (4)	0.40561 (4)	−0.00071 (4)	0.01174 (8)	
W2	−0.13497 (5)	0.63612 (4)	−0.10841 (4)	0.01522 (9)	
W3	−0.14193 (5)	0.63506 (4)	0.14178 (4)	0.01416 (9)	
W4	0.16324 (5)	0.46361 (4)	−0.00354 (4)	0.01246 (9)	
W5	0.18504 (5)	0.69819 (4)	−0.10194 (4)	0.01641 (10)	
W6	0.18457 (5)	0.93664 (4)	0.05396 (4)	0.01496 (9)	
W7	0.18302 (5)	0.93205 (4)	0.30608 (4)	0.01196 (8)	
W8	0.16177 (5)	0.67811 (4)	0.41200 (4)	0.01570 (9)	
W9	0.16303 (5)	0.45739 (4)	0.26823 (4)	0.01452 (9)	
W10	0.46736 (4)	0.73897 (4)	0.14941 (4)	0.01258 (9)	
W11	0.47530 (5)	0.98427 (4)	0.30317 (4)	0.01202 (8)	
W12	0.45930 (5)	0.73633 (4)	0.41972 (4)	0.01493 (9)	
Si1	0.1672 (4)	0.6917 (3)	0.1533 (3)	0.0103 (4)	
O1	−0.2798 (10)	0.2550 (8)	−0.0680 (9)	0.032 (2)	
O2	−0.2170 (8)	0.4662 (7)	−0.1266 (7)	0.0195 (16)	
O3	−0.2280 (8)	0.4607 (7)	0.0663 (7)	0.0214 (16)	
O4	−0.0195 (9)	0.4134 (7)	−0.0448 (7)	0.0229 (17)	
O5	−0.0245 (8)	0.4051 (7)	0.1438 (7)	0.0193 (16)	
O6	−0.2618 (11)	0.6268 (8)	−0.2387 (8)	0.025 (2)	
O7	−0.2167 (9)	0.6408 (7)	−0.0155 (6)	0.0204 (17)	
O8	−0.0031 (8)	0.6306 (7)	−0.1391 (7)	0.0223 (16)	
O9	−0.0020 (7)	0.8013 (6)	−0.0264 (6)	0.0177 (14)	
O10	−0.2741 (10)	0.6188 (9)	0.1611 (10)	0.028 (2)	
O11	−0.0091 (8)	0.7977 (6)	0.2052 (6)	0.0184 (15)	
O12	−0.0246 (8)	0.6143 (7)	0.2803 (7)	0.0207 (16)	
O13	0.1479 (10)	0.3379 (9)	−0.0669 (8)	0.028 (2)	
O14	0.1500 (8)	0.5366 (6)	−0.1235 (6)	0.0192 (16)	
O15	0.2035 (8)	0.4516 (7)	0.1496 (7)	0.0200 (16)	
O16	0.3714 (8)	0.5695 (7)	0.0732 (7)	0.0196 (16)	
O17	0.1793 (10)	0.7144 (9)	−0.2292 (8)	0.024 (2)	
O18	0.2243 (7)	0.8443 (6)	−0.0234 (6)	0.0173 (15)	
O19	0.3825 (8)	0.7551 (6)	−0.0038 (6)	0.0181 (16)	
O20	0.1779 (10)	1.0393 (9)	−0.0145 (8)	0.0222 (19)	
O21	0.1553 (8)	0.9874 (7)	0.1744 (7)	0.0195 (16)	
O22	0.3853 (8)	1.0299 (7)	0.1754 (7)	0.0176 (16)	
O23	0.1653 (10)	1.0265 (8)	0.3810 (8)	0.028 (2)	
O24	0.2077 (8)	0.8306 (6)	0.3974 (6)	0.0161 (15)	
O25	0.3809 (8)	1.0237 (7)	0.3696 (7)	0.0208 (17)	
O26	0.1411 (10)	0.6798 (8)	0.5279 (7)	0.0193 (18)	
O27	0.1308 (7)	0.5192 (6)	0.3814 (6)	0.0169 (14)	
O28	0.3611 (7)	0.7355 (6)	0.4988 (7)	0.0188 (15)	
O29	0.1464 (10)	0.3320 (9)	0.2984 (8)	0.025 (2)	
O30	0.3627 (7)	0.5630 (6)	0.3864 (6)	0.0176 (14)	
O31	0.6248 (11)	0.7802 (9)	0.1674 (10)	0.031 (2)	
O32	0.4911 (9)	0.8879 (7)	0.2113 (7)	0.0213 (16)	
O33	0.4829 (9)	0.7137 (7)	0.2913 (7)	0.0220 (17)	
O34	0.6359 (9)	1.1094 (7)	0.3846 (8)	0.0240 (19)	
O35	0.4857 (8)	0.8838 (7)	0.4038 (7)	0.0202 (16)	
O36	0.6129 (10)	0.7745 (8)	0.5385 (7)	0.0208 (18)	
O37	−0.0037 (7)	0.6157 (6)	0.0756 (6)	0.0115 (12)	
O38	0.2253 (7)	0.6572 (6)	0.0775 (6)	0.0135 (13)	
O39	0.2304 (7)	0.8335 (5)	0.1934 (6)	0.0127 (13)	
O40	0.2204 (7)	0.6525 (6)	0.2723 (5)	0.0105 (12)	
O1A	0.5034 (11)	0.4077 (7)	0.4512 (9)	0.025 (2)	
N1A	0.3843 (12)	0.2278 (10)	−0.0626 (10)	0.025 (2)	
N2A	0.4103 (12)	0.1469 (11)	−0.0912 (10)	0.027 (2)	
N3A	0.5069 (10)	0.1532 (9)	0.0118 (10)	0.017 (2)	
H3NA	0.5429	0.1081	0.0178	0.021*	
N4A	0.5432 (11)	0.2324 (9)	0.1031 (9)	0.0162 (19)	
N5A	0.5192 (10)	0.3837 (8)	0.2437 (9)	0.0143 (19)	
H5NA	0.5988	0.3824	0.2713	0.017*	
C1A	0.4675 (11)	0.2787 (9)	0.0562 (9)	0.0093 (18)	
C2A	0.4658 (13)	0.3733 (11)	0.1172 (11)	0.017 (2)	
H2AA	0.3679	0.3558	0.0779	0.020*	
H2AB	0.5253	0.4508	0.1118	0.020*	
C3A	0.5592 (13)	0.5031 (10)	0.3194 (13)	0.024 (3)	
H3AA	0.6350	0.5712	0.3166	0.029*	
H3AB	0.4759	0.5111	0.2864	0.029*	
C4A	0.6084 (13)	0.5081 (12)	0.4439 (11)	0.023 (3)	
H4AA	0.6969	0.5078	0.4793	0.028*	
H4AB	0.6290	0.5837	0.4893	0.028*	
C5A	0.4720 (17)	0.2989 (12)	0.3892 (14)	0.028 (3)	
H5AA	0.4009	0.2319	0.3975	0.034*	
H5AB	0.5587	0.2957	0.4241	0.034*	
C6A	0.4162 (13)	0.2828 (10)	0.2609 (11)	0.020 (3)	
H6AA	0.3255	0.2794	0.2243	0.024*	
H6AB	0.3979	0.2058	0.2211	0.024*	
O1B	0.8262 (10)	0.9749 (8)	−0.1436 (8)	0.0214 (18)	
N1B	0.9527 (11)	1.1682 (10)	0.3755 (9)	0.020 (2)	
N2B	0.9254 (13)	1.2496 (10)	0.3944 (10)	0.026 (2)	
N3B	0.8346 (11)	1.2448 (9)	0.2940 (8)	0.019 (2)	
H3NB	0.8022	1.2928	0.2876	0.023*	
N4B	0.7936 (10)	1.1611 (9)	0.2006 (9)	0.0156 (19)	
N5B	0.8103 (10)	1.0024 (9)	0.0613 (9)	0.0136 (18)	
H5NB	0.7339	1.0016	0.0523	0.016*	
C1B	0.8743 (12)	1.1172 (12)	0.2578 (11)	0.022 (3)	
C2B	0.8729 (15)	1.0174 (12)	0.1923 (11)	0.023 (3)	
H2BA	0.8168	0.9410	0.2012	0.028*	
H2BB	0.9709	1.0356	0.2280	0.028*	
C3B	0.7827 (12)	0.8882 (10)	−0.0014 (10)	0.015 (2)	
H3BA	0.7116	0.8196	0.0055	0.019*	
H3BB	0.8709	0.8875	0.0345	0.019*	
C4B	0.7286 (13)	0.8765 (10)	−0.1296 (11)	0.018 (2)	
H4BA	0.7115	0.8009	−0.1712	0.022*	
H4BB	0.6374	0.8723	−0.1659	0.022*	
C5B	0.8432 (17)	1.0839 (11)	−0.0878 (12)	0.026 (3)	
H5BA	0.7498	1.0756	−0.1223	0.031*	
H5BB	0.9044	1.1527	−0.1023	0.031*	
C6B	0.9093 (14)	1.1068 (12)	0.0443 (11)	0.021 (3)	
H6BA	1.0024	1.1145	0.0793	0.025*	
H6BB	0.9235	1.1821	0.0830	0.025*	
O1C	−0.1254 (9)	0.7597 (7)	0.4772 (8)	0.0220 (19)	
N1C	0.0611 (10)	1.2535 (9)	0.6738 (8)	0.0143 (18)	
N2C	0.0020 (11)	1.3170 (9)	0.6517 (9)	0.020 (2)	
N3C	−0.1102 (11)	1.2594 (9)	0.6570 (9)	0.019 (2)	
H3NC	−0.1678	1.2850	0.6452	0.023*	
N4C	−0.1311 (10)	1.1593 (9)	0.6814 (8)	0.0152 (18)	
N5C	0.0089 (10)	0.9902 (8)	0.6439 (9)	0.0151 (18)	
H5NC	0.0904	1.0345	0.6466	0.018*	
C1C	−0.0207 (13)	1.1583 (10)	0.6923 (10)	0.015 (2)	
C2C	0.0090 (13)	1.0651 (10)	0.7317 (10)	0.016 (2)	
H2CA	0.1023	1.1054	0.8063	0.019*	
H2CB	−0.0633	1.0122	0.7481	0.019*	
C3C	0.0082 (17)	0.8832 (13)	0.6814 (11)	0.030 (3)	
H3CA	0.0968	0.9126	0.7578	0.036*	
H3CB	−0.0719	0.8411	0.6928	0.036*	
C4C	−0.0047 (15)	0.7951 (13)	0.5912 (13)	0.034 (3)	
H4CA	0.0820	0.8330	0.5880	0.041*	
H4CB	−0.0146	0.7235	0.6149	0.041*	
C5C	−0.1087 (17)	0.8638 (13)	0.4395 (13)	0.030 (3)	
H5CA	−0.1856	0.8378	0.3568	0.036*	
H5CB	−0.0173	0.9060	0.4439	0.036*	
C6C	−0.1129 (12)	0.9449 (10)	0.5156 (10)	0.014 (2)	
H6CA	−0.2043	0.9022	0.5111	0.017*	
H6CB	−0.1055	1.0140	0.4878	0.017*	
O1D	0.4480 (9)	0.6171 (8)	0.8290 (8)	0.0213 (18)	
N1D	0.2558 (11)	0.1224 (10)	0.6295 (10)	0.024 (2)	
N2D	0.3111 (12)	0.0544 (10)	0.6502 (9)	0.024 (2)	
N3D	0.4246 (13)	0.1078 (11)	0.6461 (10)	0.030 (3)	
H3ND	0.4793	0.0790	0.6583	0.036*	
N4D	0.4516 (11)	0.2080 (10)	0.6224 (10)	0.024 (2)	
N5D	0.3177 (9)	0.3856 (9)	0.6646 (8)	0.0148 (18)	
H5ND	0.2344	0.3336	0.6560	0.018*	
C1D	0.3438 (12)	0.2162 (11)	0.6128 (10)	0.020 (2)	
C2D	0.3143 (14)	0.3088 (12)	0.5742 (10)	0.022 (3)	
H2DA	0.2200	0.2685	0.5007	0.027*	
H2DB	0.3852	0.3608	0.5562	0.027*	
C3D	0.3106 (16)	0.4874 (13)	0.6265 (12)	0.028 (3)	
H3DA	0.2198	0.4555	0.5512	0.034*	
H3DB	0.3883	0.5320	0.6129	0.034*	
C4D	0.3225 (14)	0.5722 (11)	0.7184 (11)	0.023 (3)	
H4DA	0.3210	0.6401	0.6918	0.028*	
H4DB	0.2399	0.5293	0.7267	0.028*	
C5D	0.4459 (13)	0.5195 (10)	0.8663 (11)	0.016 (2)	
H5DA	0.3605	0.4753	0.8706	0.020*	
H5DB	0.5297	0.5503	0.9466	0.020*	
C6D	0.4459 (12)	0.4327 (11)	0.7848 (10)	0.018 (2)	
H6DA	0.5325	0.4747	0.7819	0.021*	
H6DB	0.4440	0.3656	0.8147	0.021*	
O1W	0.5437 (8)	0.9624 (7)	−0.0360 (8)	0.0181 (17)	
H1WA	0.5098	0.9594	−0.1077	0.027*	
H1WB	0.4881	0.9048	−0.0245	0.027*	
O2W	0.7943 (9)	0.4321 (8)	0.3404 (8)	0.0198 (18)	
H2WA	0.8449	0.4362	0.4106	0.030*	
H2WB	0.8434	0.4714	0.3129	0.030*	
O3W	0.5808 (9)	0.0212 (8)	0.6389 (7)	0.0201 (17)	
H3WA	0.5521	−0.0458	0.6511	0.030*	
H3WB	0.5453	−0.0076	0.5655	0.030*	
O4W	0.7289 (10)	0.3445 (9)	0.6560 (9)	0.031 (2)	
H4WA	0.7855	0.4129	0.7095	0.046*	
H4WB	0.6594	0.3512	0.6127	0.046*	
O5W	0.4149 (10)	0.8783 (8)	0.7282 (8)	0.035 (2)	
H5WA	0.3925	0.9294	0.7094	0.052*	
H5WB	0.3548	0.8242	0.6608	0.052*	
O6W	0.9460 (12)	0.4968 (9)	0.5690 (9)	0.041 (3)	
H6WA	1.0181	0.5679	0.6054	0.061*	
H6WB	0.9624	0.4467	0.5990	0.061*	

### Atomic displacement parameters (Å^2^)

**Table:**

	*U*^11^	*U*^22^	*U*^33^	*U*^12^	*U*^13^	*U*^23^
W1	0.01137 (19)	0.00894 (18)	0.01120 (19)	0.00468 (16)	0.00413 (17)	0.00237 (16)
W2	0.0114 (2)	0.0185 (2)	0.0111 (2)	0.00985 (18)	0.00158 (16)	−0.00237 (17)
W3	0.01138 (19)	0.0226 (2)	0.0130 (2)	0.01095 (18)	0.00743 (17)	0.00836 (17)
W4	0.0188 (2)	0.01091 (19)	0.01065 (19)	0.01021 (17)	0.00775 (17)	0.00294 (16)
W5	0.0177 (2)	0.0171 (2)	0.0111 (2)	0.00503 (18)	0.00995 (18)	0.00127 (17)
W6	0.0182 (2)	0.00896 (19)	0.0224 (2)	0.00833 (18)	0.01289 (19)	0.00590 (17)
W7	0.0167 (2)	0.01184 (19)	0.00934 (19)	0.00986 (17)	0.00616 (17)	0.00235 (16)
W8	0.0148 (2)	0.0187 (2)	0.00999 (19)	0.00472 (17)	0.00866 (17)	0.00102 (16)
W9	0.0165 (2)	0.00937 (19)	0.0234 (2)	0.00780 (17)	0.01354 (19)	0.00702 (17)
W10	0.0118 (2)	0.0193 (2)	0.0125 (2)	0.01052 (17)	0.00798 (17)	0.00828 (17)
W11	0.01218 (19)	0.00813 (18)	0.01156 (19)	0.00481 (16)	0.00392 (16)	0.00277 (15)
W12	0.01094 (19)	0.0175 (2)	0.0124 (2)	0.00924 (17)	0.00247 (17)	−0.00101 (16)
Si1	0.0086 (9)	0.0107 (9)	0.0102 (9)	0.0046 (8)	0.0049 (8)	0.0007 (8)
O1	0.022 (4)	0.025 (4)	0.029 (5)	0.000 (4)	0.012 (4)	0.005 (4)
O2	0.029 (4)	0.019 (4)	0.015 (4)	0.018 (4)	0.011 (3)	0.006 (3)
O3	0.024 (4)	0.028 (4)	0.021 (4)	0.021 (4)	0.010 (3)	0.015 (3)
O4	0.025 (4)	0.037 (5)	0.020 (4)	0.021 (4)	0.017 (4)	0.015 (4)
O5	0.018 (4)	0.034 (5)	0.017 (4)	0.016 (4)	0.014 (3)	0.013 (3)
O6	0.037 (5)	0.025 (4)	0.012 (4)	0.022 (4)	0.005 (4)	0.008 (3)
O7	0.036 (5)	0.025 (4)	0.011 (4)	0.023 (4)	0.013 (4)	0.013 (3)
O8	0.025 (4)	0.021 (4)	0.024 (4)	0.010 (3)	0.018 (4)	0.003 (3)
O9	0.013 (3)	0.020 (4)	0.017 (4)	0.010 (3)	0.004 (3)	0.006 (3)
O10	0.022 (4)	0.029 (5)	0.052 (6)	0.018 (4)	0.028 (5)	0.018 (4)
O11	0.023 (4)	0.016 (3)	0.012 (3)	0.011 (3)	0.006 (3)	0.003 (3)
O12	0.024 (4)	0.024 (4)	0.019 (4)	0.014 (3)	0.014 (3)	0.012 (3)
O13	0.027 (5)	0.041 (5)	0.017 (4)	0.025 (4)	0.007 (4)	−0.001 (4)
O14	0.018 (4)	0.017 (4)	0.011 (3)	0.004 (3)	0.003 (3)	0.006 (3)
O15	0.021 (4)	0.032 (4)	0.020 (4)	0.018 (4)	0.016 (3)	0.016 (4)
O16	0.026 (4)	0.021 (4)	0.020 (4)	0.015 (3)	0.015 (3)	0.010 (3)
O17	0.018 (4)	0.031 (5)	0.012 (4)	0.006 (4)	0.008 (3)	0.000 (3)
O18	0.011 (3)	0.021 (4)	0.013 (3)	0.006 (3)	0.004 (3)	0.006 (3)
O19	0.015 (4)	0.016 (4)	0.011 (3)	0.002 (3)	0.003 (3)	0.006 (3)
O20	0.027 (5)	0.035 (5)	0.023 (4)	0.025 (4)	0.016 (4)	0.025 (4)
O21	0.026 (4)	0.029 (4)	0.012 (4)	0.021 (4)	0.009 (3)	0.012 (3)
O22	0.013 (4)	0.025 (4)	0.021 (4)	0.012 (3)	0.010 (3)	0.012 (3)
O23	0.024 (4)	0.026 (5)	0.021 (4)	0.018 (4)	0.002 (4)	−0.009 (4)
O24	0.017 (4)	0.013 (3)	0.009 (3)	0.004 (3)	0.005 (3)	0.001 (3)
O25	0.021 (4)	0.029 (4)	0.020 (4)	0.020 (4)	0.010 (3)	0.017 (4)
O26	0.034 (5)	0.022 (4)	0.009 (4)	0.018 (4)	0.013 (4)	0.011 (3)
O27	0.012 (3)	0.014 (3)	0.018 (4)	0.004 (3)	0.007 (3)	0.001 (3)
O28	0.014 (3)	0.014 (3)	0.023 (4)	0.002 (3)	0.013 (3)	−0.003 (3)
O29	0.025 (4)	0.031 (5)	0.028 (5)	0.015 (4)	0.018 (4)	0.020 (4)
O30	0.016 (3)	0.020 (4)	0.016 (4)	0.011 (3)	0.007 (3)	0.000 (3)
O31	0.044 (6)	0.024 (4)	0.056 (6)	0.026 (4)	0.038 (5)	0.026 (4)
O32	0.030 (4)	0.026 (4)	0.017 (4)	0.019 (4)	0.015 (4)	0.014 (3)
O33	0.035 (5)	0.024 (4)	0.017 (4)	0.022 (4)	0.015 (4)	0.008 (3)
O34	0.021 (4)	0.011 (4)	0.021 (4)	−0.002 (3)	0.010 (3)	−0.007 (3)
O35	0.028 (4)	0.023 (4)	0.013 (4)	0.016 (4)	0.010 (3)	0.004 (3)
O36	0.026 (4)	0.024 (4)	0.011 (4)	0.019 (4)	0.004 (3)	0.002 (3)
O37	0.008 (3)	0.016 (3)	0.010 (3)	0.007 (3)	0.004 (3)	0.005 (3)
O38	0.016 (3)	0.011 (3)	0.016 (3)	0.007 (3)	0.010 (3)	0.003 (3)
O39	0.013 (3)	0.006 (3)	0.021 (4)	0.006 (3)	0.008 (3)	0.004 (3)
O40	0.015 (3)	0.017 (3)	0.007 (3)	0.014 (3)	0.006 (3)	0.007 (3)
O1A	0.040 (5)	0.011 (4)	0.032 (5)	0.013 (4)	0.026 (5)	0.008 (4)
N1A	0.021 (5)	0.029 (6)	0.021 (5)	0.014 (5)	0.010 (5)	0.004 (4)
N2A	0.023 (5)	0.037 (6)	0.018 (5)	0.016 (5)	0.010 (5)	0.005 (5)
N3A	0.017 (5)	0.016 (5)	0.030 (6)	0.011 (4)	0.019 (5)	0.008 (4)
N4A	0.020 (5)	0.011 (4)	0.013 (5)	0.008 (4)	0.007 (4)	0.002 (4)
N5A	0.005 (4)	0.013 (4)	0.016 (5)	0.001 (4)	0.004 (4)	−0.001 (4)
C1A	0.009 (2)	0.009 (2)	0.009 (2)	0.0023 (17)	0.0074 (19)	0.0035 (17)
C2A	0.022 (6)	0.019 (6)	0.017 (6)	0.014 (5)	0.012 (5)	0.009 (5)
C3A	0.022 (6)	0.006 (5)	0.047 (8)	0.005 (4)	0.024 (6)	0.003 (5)
C4A	0.016 (6)	0.030 (7)	0.021 (6)	0.009 (5)	0.012 (5)	0.001 (5)
C5A	0.034 (7)	0.012 (6)	0.036 (8)	0.007 (5)	0.022 (6)	−0.001 (5)
C6A	0.018 (6)	0.005 (5)	0.020 (6)	−0.003 (4)	0.008 (5)	−0.005 (4)
O1B	0.028 (5)	0.018 (4)	0.019 (4)	0.009 (4)	0.016 (4)	0.002 (3)
N1B	0.020 (5)	0.030 (5)	0.013 (5)	0.014 (4)	0.008 (4)	0.008 (4)
N2B	0.040 (7)	0.034 (6)	0.026 (6)	0.026 (5)	0.027 (5)	0.016 (5)
N3B	0.022 (5)	0.023 (5)	0.006 (4)	0.012 (4)	0.003 (4)	0.006 (4)
N4B	0.014 (5)	0.021 (5)	0.016 (5)	0.009 (4)	0.011 (4)	0.007 (4)
N5B	0.017 (5)	0.016 (5)	0.018 (5)	0.015 (4)	0.010 (4)	0.010 (4)
C1B	0.012 (5)	0.032 (6)	0.019 (5)	0.015 (5)	0.003 (4)	0.007 (5)
C2B	0.027 (7)	0.021 (6)	0.018 (6)	0.015 (6)	0.007 (6)	0.008 (5)
C3B	0.016 (5)	0.022 (6)	0.017 (5)	0.014 (5)	0.011 (4)	0.010 (4)
C4B	0.020 (6)	0.007 (5)	0.024 (6)	0.006 (4)	0.011 (5)	−0.003 (4)
C5B	0.047 (8)	0.013 (6)	0.023 (6)	0.011 (6)	0.026 (6)	0.010 (5)
C6B	0.027 (6)	0.031 (6)	0.019 (6)	0.016 (5)	0.020 (5)	0.013 (5)
O1C	0.024 (5)	0.011 (4)	0.020 (4)	0.008 (4)	0.005 (4)	0.002 (3)
N1C	0.018 (5)	0.017 (4)	0.012 (4)	0.013 (4)	0.007 (4)	0.006 (4)
N2C	0.027 (5)	0.022 (5)	0.012 (4)	0.017 (4)	0.007 (4)	0.006 (4)
N3C	0.021 (5)	0.026 (5)	0.013 (5)	0.020 (4)	0.005 (4)	0.007 (4)
N4C	0.021 (5)	0.018 (4)	0.012 (4)	0.014 (4)	0.009 (4)	0.004 (4)
N5C	0.016 (4)	0.015 (4)	0.020 (5)	0.010 (4)	0.013 (4)	0.007 (4)
C1C	0.023 (6)	0.017 (5)	0.016 (5)	0.015 (5)	0.013 (5)	0.006 (4)
C2C	0.027 (6)	0.018 (5)	0.009 (5)	0.017 (5)	0.009 (5)	0.009 (4)
C3C	0.045 (8)	0.037 (7)	0.010 (5)	0.031 (7)	0.006 (6)	0.007 (5)
C4C	0.030 (7)	0.032 (7)	0.029 (7)	0.021 (6)	0.006 (6)	−0.002 (6)
C5C	0.044 (8)	0.026 (7)	0.023 (7)	0.017 (7)	0.021 (7)	0.008 (6)
C6C	0.013 (3)	0.013 (3)	0.014 (3)	0.0078 (19)	0.0058 (19)	0.0076 (19)
O1D	0.015 (4)	0.022 (4)	0.024 (5)	0.011 (4)	0.007 (4)	0.005 (4)
N1D	0.017 (5)	0.031 (6)	0.016 (5)	0.013 (5)	0.004 (4)	0.001 (4)
N2D	0.027 (5)	0.028 (5)	0.017 (5)	0.015 (5)	0.011 (4)	0.002 (4)
N3D	0.034 (6)	0.039 (6)	0.021 (5)	0.029 (5)	0.009 (5)	0.002 (5)
N4D	0.017 (5)	0.029 (5)	0.021 (5)	0.014 (4)	0.007 (4)	−0.001 (4)
N5D	0.006 (4)	0.021 (5)	0.009 (4)	0.007 (4)	−0.001 (3)	0.000 (4)
C1D	0.011 (5)	0.028 (6)	0.007 (5)	0.006 (5)	0.000 (4)	−0.005 (4)
C2D	0.023 (6)	0.038 (7)	0.015 (6)	0.023 (6)	0.010 (5)	0.005 (5)
C3D	0.041 (8)	0.035 (7)	0.022 (6)	0.028 (6)	0.018 (6)	0.022 (6)
C4D	0.023 (6)	0.023 (6)	0.023 (6)	0.018 (5)	0.005 (5)	0.011 (5)
C5D	0.015 (5)	0.010 (5)	0.015 (5)	0.006 (4)	0.002 (5)	0.000 (4)
C6D	0.013 (5)	0.024 (5)	0.013 (5)	0.011 (4)	0.003 (4)	0.010 (4)
O1W	0.011 (4)	0.018 (4)	0.018 (4)	0.001 (3)	0.009 (3)	0.000 (3)
O2W	0.019 (4)	0.022 (4)	0.027 (5)	0.015 (4)	0.013 (4)	0.011 (4)
O3W	0.016 (4)	0.025 (4)	0.014 (4)	0.012 (3)	0.003 (3)	−0.004 (3)
O4W	0.034 (5)	0.045 (6)	0.027 (5)	0.031 (5)	0.015 (4)	0.014 (4)
O5W	0.034 (5)	0.022 (4)	0.015 (4)	0.002 (4)	0.002 (4)	0.000 (3)
O6W	0.057 (7)	0.030 (5)	0.026 (5)	0.022 (5)	0.015 (5)	0.016 (4)

### Geometric parameters (Å, °)

**Table:**

W1—O1	1.722 (9)	C4A—H4AA	0.9900
W1—O5	1.880 (8)	C4A—H4AB	0.9900
W1—O3	1.886 (8)	C5A—C6A	1.50 (2)
W1—O2	1.924 (8)	C5A—H5AA	0.9900
W1—O4	1.935 (8)	C5A—H5AB	0.9900
W1—O37	2.347 (6)	C6A—H6AA	0.9900
W2—O6	1.706 (9)	C6A—H6AB	0.9900
W2—O9	1.882 (7)	O1B—C4B	1.423 (14)
W2—O8	1.890 (7)	O1B—C5B	1.454 (15)
W2—O7	1.939 (8)	N1B—N2B	1.302 (15)
W2—O2	1.946 (7)	N1B—C1B	1.341 (16)
W2—O37	2.395 (6)	N2B—N3B	1.291 (16)
W3—O10	1.701 (8)	N3B—N4B	1.339 (14)
W3—O11	1.844 (7)	N3B—H3NB	0.8800
W3—O7	1.914 (7)	N4B—C1B	1.351 (15)
W3—O12	1.918 (8)	N5B—C3B	1.484 (14)
W3—O3	1.997 (8)	N5B—C6B	1.499 (15)
W3—O37	2.351 (6)	N5B—C2B	1.527 (16)
W4—O13	1.700 (9)	N5B—H5NB	0.8999
W4—O4	1.848 (8)	C1B—C2B	1.522 (18)
W4—O14	1.912 (7)	C2B—H2BA	0.9900
W4—O15	1.921 (7)	C2B—H2BB	0.9900
W4—O16	1.986 (8)	C3B—C4B	1.508 (16)
W4—O38	2.327 (6)	C3B—H3BA	0.9900
W5—O17	1.708 (8)	C3B—H3BB	0.9900
W5—O18	1.868 (8)	C4B—H4BA	0.9900
W5—O8	1.891 (8)	C4B—H4BB	0.9900
W5—O19	1.922 (7)	C5B—C6B	1.523 (18)
W5—O14	1.946 (8)	C5B—H5BA	0.9900
W5—O38	2.382 (7)	C5B—H5BB	0.9900
W6—O20	1.697 (8)	C6B—H6BA	0.9900
W6—O9	1.889 (7)	C6B—H6BB	0.9900
W6—O18	1.903 (7)	O1C—C4C	1.433 (17)
W6—O21	1.944 (8)	O1C—C5C	1.453 (16)
W6—O22	1.947 (7)	N1C—N2C	1.311 (14)
W6—O39	2.399 (6)	N1C—C1C	1.342 (15)
W7—O23	1.697 (9)	N2C—N3C	1.298 (15)
W7—O25	1.898 (8)	N3C—N4C	1.320 (14)
W7—O21	1.904 (7)	N3C—H3NC	0.8800
W7—O24	1.906 (7)	N4C—C1C	1.323 (15)
W7—O11	1.929 (8)	N5C—C2C	1.484 (15)
W7—O39	2.351 (6)	N5C—C3C	1.526 (16)
W8—O26	1.684 (8)	N5C—C6C	1.534 (15)
W8—O24	1.880 (7)	N5C—H5NC	0.8999
W8—O12	1.907 (8)	C1C—C2C	1.498 (15)
W8—O27	1.931 (7)	C2C—H2CA	0.9900
W8—O28	1.933 (7)	C2C—H2CB	0.9900
W8—O40	2.346 (6)	C3C—C4C	1.518 (19)
W9—O29	1.673 (9)	C3C—H3CA	0.9900
W9—O15	1.876 (7)	C3C—H3CB	0.9900
W9—O5	1.915 (8)	C4C—H4CA	0.9900
W9—O30	1.938 (7)	C4C—H4CB	0.9900
W9—O27	1.947 (7)	C5C—C6C	1.461 (18)
W9—O40	2.331 (6)	C5C—H5CA	0.9900
W10—O31	1.676 (10)	C5C—H5CB	0.9900
W10—O33	1.893 (8)	C6C—H6CA	0.9900
W10—O32	1.918 (8)	C6C—H6CB	0.9900
W10—O16	1.922 (7)	O1D—C5D	1.405 (14)
W10—O19	1.927 (7)	O1D—C4D	1.412 (15)
W10—O38	2.358 (7)	N1D—N2D	1.330 (15)
W11—O34	1.671 (8)	N1D—C1D	1.351 (17)
W11—O32	1.842 (8)	N2D—N3D	1.298 (16)
W11—O22	1.894 (7)	N3D—N4D	1.309 (16)
W11—O35	1.956 (8)	N3D—H3ND	0.8800
W11—O25	1.965 (8)	N4D—C1D	1.344 (15)
W11—O39	2.375 (6)	N5D—C3D	1.492 (16)
W12—O36	1.667 (9)	N5D—C6D	1.494 (14)
W12—O35	1.866 (8)	N5D—C2D	1.499 (15)
W12—O33	1.896 (8)	N5D—H5ND	0.9001
W12—O28	1.938 (7)	C1D—C2D	1.486 (18)
W12—O30	1.950 (7)	C2D—H2DA	0.9900
W12—O40	2.353 (6)	C2D—H2DB	0.9900
Si1—O39	1.606 (7)	C3D—C4D	1.514 (19)
Si1—O37	1.616 (7)	C3D—H3DA	0.9900
Si1—O38	1.627 (7)	C3D—H3DB	0.9900
Si1—O40	1.652 (7)	C4D—H4DA	0.9900
O1A—C5A	1.398 (15)	C4D—H4DB	0.9900
O1A—C4A	1.436 (15)	C5D—C6D	1.527 (17)
N1A—N2A	1.324 (16)	C5D—H5DA	0.9900
N1A—C1A	1.353 (15)	C5D—H5DB	0.9900
N2A—N3A	1.335 (16)	C6D—H6DA	0.9900
N3A—N4A	1.307 (14)	C6D—H6DB	0.9900
N3A—H3NA	0.8800	O1W—H1WA	0.8499
N4A—C1A	1.302 (14)	O1W—H1WB	0.8499
N5A—C2A	1.491 (15)	O2W—H2WA	0.8498
N5A—C6A	1.501 (14)	O2W—H2WB	0.8501
N5A—C3A	1.547 (15)	O3W—H3WA	0.8503
N5A—H5NA	0.8999	O3W—H3WB	0.8501
C1A—C2A	1.458 (15)	O4W—H4WA	0.8500
C2A—H2AA	0.9900	O4W—H4WB	0.8501
C2A—H2AB	0.9900	O5W—H5WA	0.8499
C3A—C4A	1.485 (19)	O5W—H5WB	0.8501
C3A—H3AA	0.9900	O6W—H6WA	0.8500
C3A—H3AB	0.9900	O6W—H6WB	0.8499
			
O1—W1—O5	100.8 (4)	W1—O37—W3	92.7 (2)
O1—W1—O3	101.0 (4)	Si1—O37—W2	122.4 (3)
O5—W1—O3	92.9 (3)	W1—O37—W2	92.1 (2)
O1—W1—O2	100.5 (4)	W3—O37—W2	91.3 (2)
O5—W1—O2	158.0 (3)	Si1—O38—W4	124.9 (4)
O3—W1—O2	88.3 (3)	Si1—O38—W10	123.6 (4)
O1—W1—O4	100.7 (4)	W4—O38—W10	93.2 (2)
O5—W1—O4	84.5 (3)	Si1—O38—W5	122.3 (4)
O3—W1—O4	158.3 (3)	W4—O38—W5	92.1 (2)
O2—W1—O4	86.3 (3)	W10—O38—W5	91.7 (2)
O1—W1—O37	171.1 (4)	Si1—O39—W7	125.4 (4)
O5—W1—O37	86.6 (3)	Si1—O39—W11	124.7 (4)
O3—W1—O37	73.6 (3)	W7—O39—W11	92.3 (2)
O2—W1—O37	72.7 (3)	Si1—O39—W6	122.3 (4)
O4—W1—O37	84.8 (3)	W7—O39—W6	91.2 (2)
O6—W2—O9	105.6 (4)	W11—O39—W6	91.1 (2)
O6—W2—O8	105.4 (4)	Si1—O40—W9	124.1 (4)
O9—W2—O8	87.8 (3)	Si1—O40—W8	122.0 (3)
O6—W2—O7	98.5 (4)	W9—O40—W8	92.9 (2)
O9—W2—O7	87.6 (3)	Si1—O40—W12	124.0 (4)
O8—W2—O7	156.1 (3)	W9—O40—W12	92.9 (2)
O6—W2—O2	99.4 (4)	W8—O40—W12	92.1 (2)
O9—W2—O2	154.8 (3)	C5A—O1A—C4A	111.4 (10)
O8—W2—O2	88.1 (3)	N2A—N1A—C1A	106.8 (10)
O7—W2—O2	86.2 (3)	N1A—N2A—N3A	104.3 (10)
O6—W2—O37	166.6 (4)	N4A—N3A—N2A	114.1 (10)
O9—W2—O37	83.7 (3)	N4A—N3A—H3NA	122.9
O8—W2—O37	84.4 (3)	N2A—N3A—H3NA	122.9
O7—W2—O37	71.8 (3)	C1A—N4A—N3A	103.1 (10)
O2—W2—O37	71.2 (3)	C2A—N5A—C6A	113.0 (9)
O10—W3—O11	105.3 (4)	C2A—N5A—C3A	113.2 (9)
O10—W3—O7	99.3 (4)	C6A—N5A—C3A	107.8 (9)
O11—W3—O7	92.3 (3)	C2A—N5A—H5NA	106.2
O10—W3—O12	102.5 (4)	C6A—N5A—H5NA	109.1
O11—W3—O12	89.2 (3)	C3A—N5A—H5NA	107.3
O7—W3—O12	156.9 (3)	N4A—C1A—N1A	111.7 (10)
O10—W3—O3	96.5 (4)	N4A—C1A—C2A	127.3 (11)
O11—W3—O3	158.2 (3)	N1A—C1A—C2A	121.0 (10)
O7—W3—O3	85.5 (3)	C1A—C2A—N5A	111.6 (9)
O12—W3—O3	84.6 (3)	C1A—C2A—H2AA	109.3
O10—W3—O37	166.2 (4)	N5A—C2A—H2AA	109.3
O11—W3—O37	86.9 (3)	C1A—C2A—H2AB	109.3
O7—W3—O37	73.2 (3)	N5A—C2A—H2AB	109.3
O12—W3—O37	83.8 (3)	H2AA—C2A—H2AB	108.0
O3—W3—O37	71.7 (3)	C4A—C3A—N5A	111.3 (10)
O13—W4—O4	103.6 (4)	C4A—C3A—H3AA	109.4
O13—W4—O14	99.9 (4)	N5A—C3A—H3AA	109.4
O4—W4—O14	92.3 (3)	C4A—C3A—H3AB	109.4
O13—W4—O15	101.3 (4)	N5A—C3A—H3AB	109.4
O4—W4—O15	88.5 (3)	H3AA—C3A—H3AB	108.0
O14—W4—O15	158.0 (3)	O1A—C4A—C3A	111.0 (10)
O13—W4—O16	96.7 (4)	O1A—C4A—H4AA	109.4
O4—W4—O16	159.6 (3)	C3A—C4A—H4AA	109.4
O14—W4—O16	86.1 (3)	O1A—C4A—H4AB	109.4
O15—W4—O16	85.6 (3)	C3A—C4A—H4AB	109.4
O13—W4—O38	167.5 (4)	H4AA—C4A—H4AB	108.0
O4—W4—O38	87.4 (3)	O1A—C5A—C6A	112.2 (12)
O14—W4—O38	73.4 (3)	O1A—C5A—H5AA	109.2
O15—W4—O38	84.7 (3)	C6A—C5A—H5AA	109.2
O16—W4—O38	72.7 (3)	O1A—C5A—H5AB	109.2
O17—W5—O18	104.4 (4)	C6A—C5A—H5AB	109.2
O17—W5—O8	105.5 (4)	H5AA—C5A—H5AB	107.9
O18—W5—O8	87.6 (3)	C5A—C6A—N5A	111.2 (10)
O17—W5—O19	98.8 (4)	C5A—C6A—H6AA	109.4
O18—W5—O19	88.4 (3)	N5A—C6A—H6AA	109.4
O8—W5—O19	155.7 (3)	C5A—C6A—H6AB	109.4
O17—W5—O14	99.3 (4)	N5A—C6A—H6AB	109.4
O18—W5—O14	156.3 (3)	H6AA—C6A—H6AB	108.0
O8—W5—O14	87.2 (3)	C4B—O1B—C5B	108.2 (9)
O19—W5—O14	86.9 (3)	N2B—N1B—C1B	104.1 (10)
O17—W5—O38	166.8 (4)	N3B—N2B—N1B	108.3 (11)
O18—W5—O38	84.9 (3)	N2B—N3B—N4B	114.8 (10)
O8—W5—O38	83.9 (3)	N2B—N3B—H3NB	122.6
O19—W5—O38	71.8 (3)	N4B—N3B—H3NB	122.6
O14—W5—O38	71.5 (3)	N3B—N4B—C1B	98.5 (10)
O20—W6—O9	106.2 (4)	C3B—N5B—C6B	110.7 (9)
O20—W6—O18	106.1 (4)	C3B—N5B—C2B	109.1 (8)
O9—W6—O18	86.4 (3)	C6B—N5B—C2B	110.3 (9)
O20—W6—O21	98.6 (4)	C3B—N5B—H5NB	115.8
O9—W6—O21	89.3 (3)	C6B—N5B—H5NB	115.9
O18—W6—O21	155.1 (3)	C2B—N5B—H5NB	93.4
O20—W6—O22	98.6 (4)	N1B—C1B—N4B	114.3 (12)
O9—W6—O22	155.2 (3)	N1B—C1B—C2B	123.9 (11)
O18—W6—O22	87.7 (3)	N4B—C1B—C2B	121.9 (11)
O21—W6—O22	86.0 (3)	C1B—C2B—N5B	112.1 (10)
O20—W6—O39	166.0 (4)	C1B—C2B—H2BA	109.2
O9—W6—O39	84.2 (3)	N5B—C2B—H2BA	109.2
O18—W6—O39	83.5 (3)	C1B—C2B—H2BB	109.2
O21—W6—O39	71.7 (3)	N5B—C2B—H2BB	109.2
O22—W6—O39	71.1 (3)	H2BA—C2B—H2BB	107.9
O23—W7—O25	100.7 (4)	N5B—C3B—C4B	109.3 (9)
O23—W7—O21	98.0 (4)	N5B—C3B—H3BA	109.8
O25—W7—O21	88.8 (3)	C4B—C3B—H3BA	109.8
O23—W7—O24	104.9 (4)	N5B—C3B—H3BB	109.8
O25—W7—O24	89.8 (3)	C4B—C3B—H3BB	109.8
O21—W7—O24	156.9 (3)	H3BA—C3B—H3BB	108.3
O23—W7—O11	102.6 (4)	O1B—C4B—C3B	111.7 (10)
O25—W7—O11	156.6 (3)	O1B—C4B—H4BA	109.3
O21—W7—O11	86.9 (3)	C3B—C4B—H4BA	109.3
O24—W7—O11	85.3 (3)	O1B—C4B—H4BB	109.3
O23—W7—O39	169.0 (4)	C3B—C4B—H4BB	109.3
O25—W7—O39	72.7 (3)	H4BA—C4B—H4BB	107.9
O21—W7—O39	73.4 (3)	O1B—C5B—C6B	109.8 (11)
O24—W7—O39	84.2 (3)	O1B—C5B—H5BA	109.7
O11—W7—O39	84.1 (3)	C6B—C5B—H5BA	109.7
O26—W8—O24	104.8 (4)	O1B—C5B—H5BB	109.7
O26—W8—O12	103.5 (4)	C6B—C5B—H5BB	109.7
O24—W8—O12	88.5 (3)	H5BA—C5B—H5BB	108.2
O26—W8—O27	96.7 (4)	N5B—C6B—C5B	107.5 (10)
O24—W8—O27	158.3 (3)	N5B—C6B—H6BA	110.2
O12—W8—O27	88.8 (3)	C5B—C6B—H6BA	110.2
O26—W8—O28	97.3 (4)	N5B—C6B—H6BB	110.2
O24—W8—O28	88.9 (3)	C5B—C6B—H6BB	110.2
O12—W8—O28	159.0 (3)	H6BA—C6B—H6BB	108.5
O27—W8—O28	86.0 (3)	C4C—O1C—C5C	109.9 (10)
O26—W8—O40	165.8 (3)	N2C—N1C—C1C	106.4 (9)
O24—W8—O40	85.8 (3)	N3C—N2C—N1C	105.8 (10)
O12—W8—O40	85.9 (3)	N2C—N3C—N4C	114.6 (10)
O27—W8—O40	72.6 (3)	N2C—N3C—H3NC	122.7
O28—W8—O40	73.2 (3)	N4C—N3C—H3NC	122.7
O29—W9—O15	102.3 (4)	N3C—N4C—C1C	101.4 (9)
O29—W9—O5	103.3 (4)	C2C—N5C—C3C	108.3 (9)
O15—W9—O5	86.1 (3)	C2C—N5C—C6C	115.9 (8)
O29—W9—O30	96.7 (4)	C3C—N5C—C6C	109.3 (9)
O15—W9—O30	90.7 (3)	C2C—N5C—H5NC	107.7
O5—W9—O30	160.0 (3)	C3C—N5C—H5NC	107.6
O29—W9—O27	99.0 (4)	C6C—N5C—H5NC	107.6
O15—W9—O27	158.7 (3)	N4C—C1C—N1C	111.7 (10)
O5—W9—O27	89.9 (3)	N4C—C1C—C2C	121.1 (10)
O30—W9—O27	85.9 (3)	N1C—C1C—C2C	127.0 (10)
O29—W9—O40	167.0 (4)	N5C—C2C—C1C	113.4 (9)
O15—W9—O40	86.2 (3)	N5C—C2C—H2CA	108.9
O5—W9—O40	86.9 (3)	C1C—C2C—H2CA	108.9
O30—W9—O40	73.1 (3)	N5C—C2C—H2CB	108.9
O27—W9—O40	72.7 (3)	C1C—C2C—H2CB	108.9
O31—W10—O33	105.3 (4)	H2CA—C2C—H2CB	107.7
O31—W10—O32	103.6 (4)	C4C—C3C—N5C	112.3 (11)
O33—W10—O32	85.2 (3)	C4C—C3C—H3CA	109.1
O31—W10—O16	98.4 (4)	N5C—C3C—H3CA	109.1
O33—W10—O16	90.9 (3)	C4C—C3C—H3CB	109.1
O32—W10—O16	157.9 (3)	N5C—C3C—H3CB	109.1
O31—W10—O19	97.3 (4)	H3CA—C3C—H3CB	107.9
O33—W10—O19	157.2 (3)	O1C—C4C—C3C	110.2 (11)
O32—W10—O19	86.7 (3)	O1C—C4C—H4CA	109.6
O16—W10—O19	88.6 (3)	C3C—C4C—H4CA	109.6
O31—W10—O38	166.3 (4)	O1C—C4C—H4CB	109.6
O33—W10—O38	85.8 (3)	C3C—C4C—H4CB	109.6
O32—W10—O38	85.0 (3)	H4CA—C4C—H4CB	108.1
O16—W10—O38	73.0 (3)	O1C—C5C—C6C	109.6 (10)
O19—W10—O38	72.3 (3)	O1C—C5C—H5CA	109.8
O34—W11—O32	105.7 (4)	C6C—C5C—H5CA	109.8
O34—W11—O22	101.4 (4)	O1C—C5C—H5CB	109.8
O32—W11—O22	92.0 (3)	C6C—C5C—H5CB	109.8
O34—W11—O35	102.0 (4)	H5CA—C5C—H5CB	108.2
O32—W11—O35	86.9 (3)	C5C—C6C—N5C	110.8 (10)
O22—W11—O35	156.0 (3)	C5C—C6C—H6CA	109.5
O34—W11—O25	98.4 (4)	N5C—C6C—H6CA	109.5
O32—W11—O25	155.8 (3)	C5C—C6C—H6CB	109.5
O22—W11—O25	86.1 (3)	N5C—C6C—H6CB	109.5
O35—W11—O25	85.2 (3)	H6CA—C6C—H6CB	108.1
O34—W11—O39	167.9 (3)	C5D—O1D—C4D	108.2 (9)
O32—W11—O39	85.3 (3)	N2D—N1D—C1D	104.8 (10)
O22—W11—O39	72.5 (3)	N3D—N2D—N1D	106.9 (11)
O35—W11—O39	83.5 (3)	N2D—N3D—N4D	114.8 (11)
O25—W11—O39	71.1 (3)	N2D—N3D—H3ND	122.6
O36—W12—O35	103.3 (4)	N4D—N3D—H3ND	122.6
O36—W12—O33	104.6 (4)	N3D—N4D—C1D	101.5 (11)
O35—W12—O33	87.2 (3)	C3D—N5D—C6D	110.0 (10)
O36—W12—O28	97.9 (4)	C3D—N5D—C2D	109.2 (9)
O35—W12—O28	89.9 (3)	C6D—N5D—C2D	112.9 (9)
O33—W12—O28	157.4 (3)	C3D—N5D—H5ND	108.6
O36—W12—O30	98.1 (4)	C6D—N5D—H5ND	116.0
O35—W12—O30	158.6 (3)	C2D—N5D—H5ND	99.7
O33—W12—O30	88.4 (3)	N4D—C1D—N1D	112.0 (12)
O28—W12—O30	86.1 (3)	N4D—C1D—C2D	124.2 (11)
O36—W12—O40	166.9 (4)	N1D—C1D—C2D	123.4 (11)
O35—W12—O40	86.3 (3)	C1D—C2D—N5D	113.2 (10)
O33—W12—O40	84.5 (3)	C1D—C2D—H2DA	108.9
O28—W12—O40	72.9 (3)	N5D—C2D—H2DA	108.9
O30—W12—O40	72.4 (3)	C1D—C2D—H2DB	108.9
O39—Si1—O37	111.4 (4)	N5D—C2D—H2DB	108.9
O39—Si1—O38	111.2 (4)	H2DA—C2D—H2DB	107.7
O37—Si1—O38	109.1 (4)	N5D—C3D—C4D	111.3 (10)
O39—Si1—O40	108.8 (4)	N5D—C3D—H3DA	109.4
O37—Si1—O40	108.9 (4)	C4D—C3D—H3DA	109.4
O38—Si1—O40	107.4 (4)	N5D—C3D—H3DB	109.4
W1—O2—W2	123.9 (4)	C4D—C3D—H3DB	109.4
W1—O3—W3	122.1 (4)	H3DA—C3D—H3DB	108.0
W4—O4—W1	149.9 (5)	O1D—C4D—C3D	111.7 (10)
W1—O5—W9	151.5 (4)	O1D—C4D—H4DA	109.3
W3—O7—W2	123.5 (4)	C3D—C4D—H4DA	109.3
W2—O8—W5	149.9 (4)	O1D—C4D—H4DB	109.3
W2—O9—W6	151.8 (4)	C3D—C4D—H4DB	109.3
W3—O11—W7	151.4 (4)	H4DA—C4D—H4DB	107.9
W8—O12—W3	146.4 (4)	O1D—C5D—C6D	113.2 (10)
W4—O14—W5	123.0 (4)	O1D—C5D—H5DA	108.9
W9—O15—W4	148.0 (4)	C6D—C5D—H5DA	108.9
W10—O16—W4	121.1 (4)	O1D—C5D—H5DB	108.9
W5—O18—W6	152.0 (4)	C6D—C5D—H5DB	108.9
W5—O19—W10	124.2 (4)	H5DA—C5D—H5DB	107.8
W7—O21—W6	123.7 (4)	N5D—C6D—C5D	108.2 (9)
W11—O22—W6	125.1 (4)	N5D—C6D—H6DA	110.0
W8—O24—W7	151.6 (4)	C5D—C6D—H6DA	110.0
W7—O25—W11	123.9 (4)	N5D—C6D—H6DB	110.0
W8—O27—W9	121.9 (4)	C5D—C6D—H6DB	110.0
W8—O28—W12	121.8 (4)	H6DA—C6D—H6DB	108.4
W9—O30—W12	121.6 (4)	H1WA—O1W—H1WB	114.2
W11—O32—W10	152.7 (4)	H2WA—O2W—H2WB	112.5
W10—O33—W12	151.3 (4)	H3WA—O3W—H3WB	96.4
W12—O35—W11	148.6 (4)	H4WA—O4W—H4WB	101.1
Si1—O37—W1	125.8 (4)	H5WA—O5W—H5WB	96.0
Si1—O37—W3	123.2 (4)	H6WA—O6W—H6WB	111.9
			
O1—W1—O2—W2	172.1 (5)	O3—W3—O37—Si1	−136.3 (5)
O5—W1—O2—W2	−22.4 (11)	O10—W3—O37—W1	−31.2 (16)
O3—W1—O2—W2	71.2 (5)	O11—W3—O37—W1	176.5 (3)
O4—W1—O2—W2	−87.7 (5)	O7—W3—O37—W1	−90.2 (3)
O37—W1—O2—W2	−2.1 (4)	O12—W3—O37—W1	86.9 (3)
O6—W2—O2—W1	−168.0 (5)	O3—W3—O37—W1	0.6 (2)
O9—W2—O2—W1	5.9 (10)	O10—W3—O37—W2	61.0 (16)
O8—W2—O2—W1	86.8 (5)	O11—W3—O37—W2	−91.3 (3)
O7—W2—O2—W1	−70.0 (5)	O7—W3—O37—W2	2.0 (3)
O37—W2—O2—W1	2.1 (4)	O12—W3—O37—W2	179.2 (3)
O1—W1—O3—W3	−172.0 (5)	O3—W3—O37—W2	92.8 (3)
O5—W1—O3—W3	86.4 (5)	O6—W2—O37—Si1	−178.4 (14)
O2—W1—O3—W3	−71.6 (5)	O9—W2—O37—Si1	−44.0 (4)
O4—W1—O3—W3	4.0 (11)	O8—W2—O37—Si1	44.5 (5)
O37—W1—O3—W3	0.8 (4)	O7—W2—O37—Si1	−133.5 (5)
O10—W3—O3—W1	171.9 (5)	O2—W2—O37—Si1	134.4 (5)
O11—W3—O3—W1	−11.8 (11)	O6—W2—O37—W1	45.8 (15)
O7—W3—O3—W1	73.0 (5)	O9—W2—O37—W1	−179.8 (3)
O12—W3—O3—W1	−86.1 (5)	O8—W2—O37—W1	−91.4 (3)
O37—W3—O3—W1	−0.8 (4)	O7—W2—O37—W1	90.7 (3)
O13—W4—O4—W1	131.8 (9)	O2—W2—O37—W1	−1.4 (3)
O14—W4—O4—W1	−127.4 (9)	O6—W2—O37—W3	−46.9 (15)
O15—W4—O4—W1	30.6 (9)	O9—W2—O37—W3	87.5 (3)
O16—W4—O4—W1	−42.4 (16)	O8—W2—O37—W3	175.9 (3)
O38—W4—O4—W1	−54.2 (9)	O7—W2—O37—W3	−2.0 (3)
O1—W1—O4—W4	−132.1 (10)	O2—W2—O37—W3	−94.1 (3)
O5—W1—O4—W4	−32.1 (9)	O39—Si1—O38—W4	−179.0 (4)
O3—W1—O4—W4	51.9 (15)	O37—Si1—O38—W4	−55.9 (6)
O2—W1—O4—W4	127.9 (9)	O40—Si1—O38—W4	62.0 (5)
O37—W1—O4—W4	55.0 (9)	O39—Si1—O38—W10	58.0 (5)
O1—W1—O5—W9	137.2 (10)	O37—Si1—O38—W10	−178.8 (4)
O3—W1—O5—W9	−121.0 (10)	O40—Si1—O38—W10	−60.9 (5)
O2—W1—O5—W9	−28.3 (16)	O39—Si1—O38—W5	−59.5 (5)
O4—W1—O5—W9	37.4 (10)	O37—Si1—O38—W5	63.7 (5)
O37—W1—O5—W9	−47.7 (9)	O40—Si1—O38—W5	−178.5 (4)
O29—W9—O5—W1	−139.0 (10)	O13—W4—O38—Si1	−167.2 (16)
O15—W9—O5—W1	−37.3 (10)	O4—W4—O38—Si1	40.9 (5)
O30—W9—O5—W1	44.0 (15)	O14—W4—O38—Si1	134.1 (5)
O27—W9—O5—W1	121.7 (10)	O15—W4—O38—Si1	−47.8 (5)
O40—W9—O5—W1	49.1 (9)	O16—W4—O38—Si1	−134.9 (5)
O10—W3—O7—W2	−171.0 (5)	O13—W4—O38—W10	−31.6 (18)
O11—W3—O7—W2	83.1 (5)	O4—W4—O38—W10	176.5 (3)
O12—W3—O7—W2	−10.2 (12)	O14—W4—O38—W10	−90.3 (3)
O3—W3—O7—W2	−75.2 (5)	O15—W4—O38—W10	87.8 (3)
O37—W3—O7—W2	−3.0 (4)	O16—W4—O38—W10	0.8 (2)
O6—W2—O7—W3	173.4 (5)	O13—W4—O38—W5	60.2 (18)
O9—W2—O7—W3	−81.2 (5)	O4—W4—O38—W5	−91.7 (3)
O8—W2—O7—W3	−2.2 (11)	O14—W4—O38—W5	1.5 (3)
O2—W2—O7—W3	74.4 (5)	O15—W4—O38—W5	179.6 (3)
O37—W2—O7—W3	2.9 (4)	O16—W4—O38—W5	92.6 (3)
O6—W2—O8—W5	125.8 (9)	O31—W10—O38—Si1	−171.8 (13)
O9—W2—O8—W5	20.3 (9)	O33—W10—O38—Si1	43.4 (5)
O7—W2—O8—W5	−58.7 (14)	O32—W10—O38—Si1	−42.1 (5)
O2—W2—O8—W5	−134.9 (9)	O16—W10—O38—Si1	135.6 (5)
O37—W2—O8—W5	−63.6 (9)	O19—W10—O38—Si1	−130.3 (5)
O17—W5—O8—W2	−124.9 (9)	O31—W10—O38—W4	51.8 (15)
O18—W5—O8—W2	−20.6 (9)	O33—W10—O38—W4	−93.0 (3)
O19—W5—O8—W2	60.1 (14)	O32—W10—O38—W4	−178.5 (3)
O14—W5—O8—W2	136.2 (9)	O16—W10—O38—W4	−0.8 (3)
O38—W5—O8—W2	64.5 (9)	O19—W10—O38—W4	93.3 (3)
O6—W2—O9—W6	−125.4 (9)	O31—W10—O38—W5	−40.4 (15)
O8—W2—O9—W6	−20.1 (9)	O33—W10—O38—W5	174.8 (3)
O7—W2—O9—W6	136.5 (9)	O32—W10—O38—W5	89.3 (3)
O2—W2—O9—W6	60.8 (13)	O16—W10—O38—W5	−93.0 (3)
O37—W2—O9—W6	64.5 (9)	O19—W10—O38—W5	1.1 (3)
O20—W6—O9—W2	125.6 (9)	O17—W5—O38—Si1	177.0 (14)
O18—W6—O9—W2	19.9 (9)	O18—W5—O38—Si1	41.3 (4)
O21—W6—O9—W2	−135.6 (9)	O8—W5—O38—Si1	−46.9 (5)
O22—W6—O9—W2	−56.7 (13)	O19—W5—O38—Si1	131.2 (5)
O39—W6—O9—W2	−63.9 (9)	O14—W5—O38—Si1	−135.9 (5)
O10—W3—O11—W7	130.3 (9)	O17—W5—O38—W4	−48.6 (16)
O7—W3—O11—W7	−129.4 (9)	O18—W5—O38—W4	175.7 (3)
O12—W3—O11—W7	27.5 (9)	O8—W5—O38—W4	87.5 (3)
O3—W3—O11—W7	−45.8 (14)	O19—W5—O38—W4	−94.4 (3)
O37—W3—O11—W7	−56.3 (9)	O14—W5—O38—W4	−1.5 (3)
O23—W7—O11—W3	−132.1 (9)	O17—W5—O38—W10	44.6 (16)
O25—W7—O11—W3	50.8 (14)	O18—W5—O38—W10	−91.1 (3)
O21—W7—O11—W3	130.5 (9)	O8—W5—O38—W10	−179.2 (3)
O24—W7—O11—W3	−27.8 (9)	O19—W5—O38—W10	−1.1 (3)
O39—W7—O11—W3	56.8 (9)	O14—W5—O38—W10	91.7 (3)
O26—W8—O12—W3	127.6 (8)	O37—Si1—O39—W7	58.7 (5)
O24—W8—O12—W3	22.8 (8)	O38—Si1—O39—W7	−179.4 (4)
O27—W8—O12—W3	−135.7 (8)	O40—Si1—O39—W7	−61.3 (5)
O28—W8—O12—W3	−60.2 (14)	O37—Si1—O39—W11	−177.8 (4)
O40—W8—O12—W3	−63.1 (8)	O38—Si1—O39—W11	−55.9 (6)
O10—W3—O12—W8	−128.3 (8)	O40—Si1—O39—W11	62.2 (5)
O11—W3—O12—W8	−22.8 (8)	O37—Si1—O39—W6	−59.9 (5)
O7—W3—O12—W8	71.1 (13)	O38—Si1—O39—W6	61.9 (5)
O3—W3—O12—W8	136.2 (8)	O40—Si1—O39—W6	−179.9 (3)
O37—W3—O12—W8	64.2 (8)	O23—W7—O39—Si1	−170.9 (18)
O13—W4—O14—W5	−171.4 (5)	O25—W7—O39—Si1	134.8 (5)
O4—W4—O14—W5	84.3 (5)	O21—W7—O39—Si1	−131.2 (5)
O15—W4—O14—W5	−7.3 (12)	O24—W7—O39—Si1	43.1 (5)
O16—W4—O14—W5	−75.3 (5)	O11—W7—O39—Si1	−42.7 (5)
O38—W4—O14—W5	−2.2 (4)	O23—W7—O39—W11	52.4 (19)
O17—W5—O14—W4	172.4 (5)	O25—W7—O39—W11	−1.9 (3)
O18—W5—O14—W4	−4.8 (11)	O21—W7—O39—W11	92.1 (3)
O8—W5—O14—W4	−82.3 (5)	O24—W7—O39—W11	−93.6 (3)
O19—W5—O14—W4	74.0 (5)	O11—W7—O39—W11	−179.4 (3)
O38—W5—O14—W4	2.2 (4)	O23—W7—O39—W6	−39 (2)
O29—W9—O15—W4	131.2 (9)	O25—W7—O39—W6	−93.1 (3)
O5—W9—O15—W4	28.4 (9)	O21—W7—O39—W6	0.9 (3)
O30—W9—O15—W4	−131.8 (9)	O24—W7—O39—W6	175.2 (3)
O27—W9—O15—W4	−51.3 (15)	O11—W7—O39—W6	89.4 (3)
O40—W9—O15—W4	−58.8 (9)	O34—W11—O39—Si1	−165.9 (15)
O13—W4—O15—W9	−131.4 (9)	O32—W11—O39—Si1	39.2 (5)
O4—W4—O15—W9	−27.8 (9)	O22—W11—O39—Si1	132.8 (5)
O14—W4—O15—W9	64.7 (14)	O35—W11—O39—Si1	−48.2 (5)
O16—W4—O15—W9	132.7 (9)	O25—W11—O39—Si1	−135.3 (5)
O38—W4—O15—W9	59.7 (9)	O34—W11—O39—W7	−28.7 (17)
O31—W10—O16—W4	−168.0 (5)	O32—W11—O39—W7	176.4 (3)
O33—W10—O16—W4	86.4 (4)	O22—W11—O39—W7	−90.0 (3)
O32—W10—O16—W4	7.0 (11)	O35—W11—O39—W7	89.0 (3)
O19—W10—O16—W4	−70.8 (4)	O25—W11—O39—W7	1.9 (2)
O38—W10—O16—W4	1.1 (3)	O34—W11—O39—W6	62.5 (17)
O13—W4—O16—W10	172.2 (5)	O32—W11—O39—W6	−92.4 (3)
O4—W4—O16—W10	−13.4 (12)	O22—W11—O39—W6	1.2 (3)
O14—W4—O16—W10	72.7 (4)	O35—W11—O39—W6	−179.8 (3)
O15—W4—O16—W10	−86.9 (4)	O25—W11—O39—W6	93.1 (3)
O38—W4—O16—W10	−1.1 (4)	O20—W6—O39—Si1	−178.9 (13)
O17—W5—O18—W6	127.0 (9)	O9—W6—O39—Si1	42.3 (4)
O8—W5—O18—W6	21.6 (9)	O18—W6—O39—Si1	−44.7 (4)
O19—W5—O18—W6	−134.4 (9)	O21—W6—O39—Si1	133.4 (5)
O14—W5—O18—W6	−55.8 (13)	O22—W6—O39—Si1	−134.5 (5)
O38—W5—O18—W6	−62.5 (9)	O20—W6—O39—W7	46.8 (15)
O20—W6—O18—W5	−127.0 (9)	O9—W6—O39—W7	−92.1 (3)
O9—W6—O18—W5	−21.1 (9)	O18—W6—O39—W7	−179.0 (3)
O21—W6—O18—W5	59.3 (13)	O21—W6—O39—W7	−0.9 (3)
O22—W6—O18—W5	134.7 (9)	O22—W6—O39—W7	91.1 (3)
O39—W6—O18—W5	63.5 (9)	O20—W6—O39—W11	−45.6 (15)
O17—W5—O19—W10	−168.8 (5)	O9—W6—O39—W11	175.6 (3)
O18—W5—O19—W10	86.9 (5)	O18—W6—O39—W11	88.6 (3)
O8—W5—O19—W10	6.3 (11)	O21—W6—O39—W11	−93.2 (3)
O14—W5—O19—W10	−69.9 (5)	O22—W6—O39—W11	−1.2 (3)
O38—W5—O19—W10	1.7 (4)	O39—Si1—O40—W9	−179.4 (4)
O31—W10—O19—W5	169.2 (5)	O37—Si1—O40—W9	59.1 (5)
O33—W10—O19—W5	−18.2 (11)	O38—Si1—O40—W9	−58.9 (5)
O32—W10—O19—W5	−87.5 (5)	O39—Si1—O40—W8	60.9 (5)
O16—W10—O19—W5	70.9 (5)	O37—Si1—O40—W8	−60.6 (5)
O38—W10—O19—W5	−1.7 (4)	O38—Si1—O40—W8	−178.6 (4)
O23—W7—O21—W6	171.5 (5)	O39—Si1—O40—W12	−57.4 (5)
O25—W7—O21—W6	70.9 (5)	O37—Si1—O40—W12	−178.9 (4)
O24—W7—O21—W6	−15.9 (11)	O38—Si1—O40—W12	63.1 (5)
O11—W7—O21—W6	−86.1 (5)	O29—W9—O40—Si1	175.2 (14)
O39—W7—O21—W6	−1.4 (4)	O15—W9—O40—Si1	43.9 (4)
O20—W6—O21—W7	−168.2 (5)	O5—W9—O40—Si1	−42.4 (4)
O9—W6—O21—W7	85.5 (5)	O30—W9—O40—Si1	135.8 (5)
O18—W6—O21—W7	5.7 (11)	O27—W9—O40—Si1	−133.3 (5)
O22—W6—O21—W7	−70.1 (5)	O29—W9—O40—W8	−52.2 (16)
O39—W6—O21—W7	1.3 (4)	O15—W9—O40—W8	176.4 (3)
O34—W11—O22—W6	−171.0 (5)	O5—W9—O40—W8	90.1 (3)
O32—W11—O22—W6	82.6 (5)	O30—W9—O40—W8	−91.7 (3)
O35—W11—O22—W6	−4.3 (11)	O27—W9—O40—W8	−0.7 (2)
O25—W11—O22—W6	−73.2 (5)	O29—W9—O40—W12	40.0 (16)
O39—W11—O22—W6	−1.8 (4)	O15—W9—O40—W12	−91.4 (3)
O20—W6—O22—W11	171.9 (5)	O5—W9—O40—W12	−177.6 (3)
O9—W6—O22—W11	−5.8 (11)	O30—W9—O40—W12	0.5 (2)
O18—W6—O22—W11	−82.1 (5)	O27—W9—O40—W12	91.5 (3)
O21—W6—O22—W11	73.8 (5)	O26—W8—O40—Si1	176.9 (13)
O39—W6—O22—W11	1.8 (4)	O24—W8—O40—Si1	−44.2 (4)
O26—W8—O24—W7	−131.7 (9)	O12—W8—O40—Si1	44.6 (4)
O12—W8—O24—W7	−28.2 (9)	O27—W8—O40—Si1	134.7 (5)
O27—W8—O24—W7	54.9 (14)	O28—W8—O40—Si1	−134.3 (5)
O28—W8—O24—W7	131.0 (9)	O26—W8—O40—W9	42.9 (15)
O40—W8—O24—W7	57.8 (9)	O24—W8—O40—W9	−178.1 (3)
O23—W7—O24—W8	130.1 (9)	O12—W8—O40—W9	−89.3 (3)
O25—W7—O24—W8	−128.9 (9)	O27—W8—O40—W9	0.7 (2)
O21—W7—O24—W8	−42.3 (14)	O28—W8—O40—W9	91.8 (3)
O11—W7—O24—W8	28.2 (9)	O26—W8—O40—W12	−50.0 (15)
O39—W7—O24—W8	−56.3 (9)	O24—W8—O40—W12	88.9 (3)
O23—W7—O25—W11	−168.1 (5)	O12—W8—O40—W12	177.7 (3)
O21—W7—O25—W11	−70.2 (5)	O27—W8—O40—W12	−92.2 (3)
O24—W7—O25—W11	86.7 (5)	O28—W8—O40—W12	−1.2 (3)
O11—W7—O25—W11	9.1 (11)	O36—W12—O40—Si1	179.5 (14)
O39—W7—O25—W11	2.8 (4)	O35—W12—O40—Si1	41.8 (4)
O34—W11—O25—W7	171.0 (5)	O33—W12—O40—Si1	−45.7 (4)
O32—W11—O25—W7	−16.1 (10)	O28—W12—O40—Si1	132.9 (5)
O22—W11—O25—W7	70.1 (5)	O30—W12—O40—Si1	−135.8 (5)
O35—W11—O25—W7	−87.5 (5)	O36—W12—O40—W9	−45.2 (15)
O39—W11—O25—W7	−2.8 (4)	O35—W12—O40—W9	177.1 (3)
O26—W8—O27—W9	−171.5 (5)	O33—W12—O40—W9	89.6 (3)
O24—W8—O27—W9	2.0 (11)	O28—W12—O40—W9	−91.8 (3)
O12—W8—O27—W9	85.0 (4)	O30—W12—O40—W9	−0.5 (2)
O28—W8—O27—W9	−74.6 (4)	O36—W12—O40—W8	47.8 (15)
O40—W8—O27—W9	−1.0 (3)	O35—W12—O40—W8	−89.9 (3)
O29—W9—O27—W8	170.8 (5)	O33—W12—O40—W8	−177.4 (3)
O15—W9—O27—W8	−6.8 (11)	O28—W12—O40—W8	1.2 (3)
O5—W9—O27—W8	−85.8 (4)	O30—W12—O40—W8	92.5 (3)
O30—W9—O27—W8	74.6 (4)	C1A—N1A—N2A—N3A	0.3 (13)
O40—W9—O27—W8	1.0 (3)	N1A—N2A—N3A—N4A	0.1 (13)
O26—W8—O28—W12	171.0 (5)	N2A—N3A—N4A—C1A	−0.4 (12)
O24—W8—O28—W12	−84.2 (4)	N3A—N4A—C1A—N1A	0.6 (12)
O12—W8—O28—W12	−1.4 (12)	N3A—N4A—C1A—C2A	−179.4 (10)
O27—W8—O28—W12	74.7 (4)	N2A—N1A—C1A—N4A	−0.6 (13)
O40—W8—O28—W12	1.7 (4)	N2A—N1A—C1A—C2A	179.4 (10)
O36—W12—O28—W8	−172.1 (5)	N4A—C1A—C2A—N5A	−21.5 (16)
O35—W12—O28—W8	84.5 (5)	N1A—C1A—C2A—N5A	158.5 (10)
O33—W12—O28—W8	1.9 (11)	C6A—N5A—C2A—C1A	−73.3 (12)
O30—W12—O28—W8	−74.5 (4)	C3A—N5A—C2A—C1A	163.7 (9)
O40—W12—O28—W8	−1.7 (4)	C2A—N5A—C3A—C4A	178.6 (10)
O29—W9—O30—W12	−172.5 (5)	C6A—N5A—C3A—C4A	52.8 (12)
O15—W9—O30—W12	85.1 (4)	C5A—O1A—C4A—C3A	59.7 (14)
O5—W9—O30—W12	4.6 (11)	N5A—C3A—C4A—O1A	−56.6 (13)
O27—W9—O30—W12	−73.8 (4)	C4A—O1A—C5A—C6A	−60.0 (15)
O40—W9—O30—W12	−0.7 (3)	O1A—C5A—C6A—N5A	57.6 (15)
O36—W12—O30—W9	171.5 (5)	C2A—N5A—C6A—C5A	−178.1 (10)
O35—W12—O30—W9	−5.8 (11)	C3A—N5A—C6A—C5A	−52.2 (13)
O33—W12—O30—W9	−84.0 (4)	C1B—N1B—N2B—N3B	1.1 (14)
O28—W12—O30—W9	74.0 (4)	N1B—N2B—N3B—N4B	0.1 (14)
O40—W12—O30—W9	0.7 (3)	N2B—N3B—N4B—C1B	−1.2 (13)
O34—W11—O32—W10	128.7 (10)	N2B—N1B—C1B—N4B	−2.0 (15)
O22—W11—O32—W10	−128.9 (10)	N2B—N1B—C1B—C2B	178.7 (12)
O35—W11—O32—W10	27.1 (10)	N3B—N4B—C1B—N1B	2.0 (13)
O25—W11—O32—W10	−44.0 (15)	N3B—N4B—C1B—C2B	−178.8 (11)
O39—W11—O32—W10	−56.7 (10)	N1B—C1B—C2B—N5B	−163.2 (12)
O31—W10—O32—W11	−132.5 (10)	N4B—C1B—C2B—N5B	17.6 (17)
O33—W10—O32—W11	−27.9 (10)	C3B—N5B—C2B—C1B	−167.5 (10)
O16—W10—O32—W11	52.6 (15)	C6B—N5B—C2B—C1B	70.6 (13)
O19—W10—O32—W11	130.8 (10)	C6B—N5B—C3B—C4B	−55.2 (12)
O38—W10—O32—W11	58.3 (10)	C2B—N5B—C3B—C4B	−176.8 (10)
O31—W10—O33—W12	132.0 (10)	C5B—O1B—C4B—C3B	−62.3 (12)
O32—W10—O33—W12	29.3 (10)	N5B—C3B—C4B—O1B	58.1 (12)
O16—W10—O33—W12	−129.0 (10)	C4B—O1B—C5B—C6B	64.1 (13)
O19—W10—O33—W12	−40.3 (16)	C3B—N5B—C6B—C5B	57.0 (13)
O38—W10—O33—W12	−56.1 (10)	C2B—N5B—C6B—C5B	177.9 (10)
O36—W12—O33—W10	−132.7 (10)	O1B—C5B—C6B—N5B	−61.4 (13)
O35—W12—O33—W10	−29.7 (10)	C1C—N1C—N2C—N3C	−0.2 (12)
O28—W12—O33—W10	53.4 (15)	N1C—N2C—N3C—N4C	−0.1 (13)
O30—W12—O33—W10	129.3 (10)	N2C—N3C—N4C—C1C	0.4 (13)
O40—W12—O33—W10	56.8 (10)	N3C—N4C—C1C—N1C	−0.6 (13)
O36—W12—O35—W11	129.3 (9)	N3C—N4C—C1C—C2C	174.2 (10)
O33—W12—O35—W11	25.0 (9)	N2C—N1C—C1C—N4C	0.5 (13)
O28—W12—O35—W11	−132.6 (9)	N2C—N1C—C1C—C2C	−173.9 (11)
O30—W12—O35—W11	−53.5 (14)	C3C—N5C—C2C—C1C	−168.0 (11)
O40—W12—O35—W11	−59.7 (9)	C6C—N5C—C2C—C1C	−44.7 (13)
O34—W11—O35—W12	−129.7 (9)	N4C—C1C—C2C—N5C	119.9 (12)
O32—W11—O35—W12	−24.3 (9)	N1C—C1C—C2C—N5C	−66.1 (15)
O22—W11—O35—W12	63.6 (13)	C2C—N5C—C3C—C4C	175.0 (11)
O25—W11—O35—W12	132.7 (9)	C6C—N5C—C3C—C4C	47.8 (14)
O39—W11—O35—W12	61.3 (9)	C5C—O1C—C4C—C3C	61.7 (15)
O39—Si1—O37—W1	−178.0 (4)	N5C—C3C—C4C—O1C	−53.5 (16)
O38—Si1—O37—W1	58.9 (5)	C4C—O1C—C5C—C6C	−66.9 (14)
O40—Si1—O37—W1	−58.1 (5)	O1C—C5C—C6C—N5C	61.5 (14)
O39—Si1—O37—W3	−55.3 (5)	C2C—N5C—C6C—C5C	−174.9 (10)
O38—Si1—O37—W3	−178.4 (4)	C3C—N5C—C6C—C5C	−52.1 (13)
O40—Si1—O37—W3	64.6 (5)	C1D—N1D—N2D—N3D	0.5 (13)
O39—Si1—O37—W2	61.2 (5)	N1D—N2D—N3D—N4D	−0.9 (15)
O38—Si1—O37—W2	−61.9 (5)	N2D—N3D—N4D—C1D	0.9 (14)
O40—Si1—O37—W2	−178.8 (3)	N3D—N4D—C1D—N1D	−0.5 (13)
O5—W1—O37—Si1	40.5 (5)	N3D—N4D—C1D—C2D	−173.6 (11)
O3—W1—O37—Si1	134.6 (5)	N2D—N1D—C1D—N4D	0.1 (14)
O2—W1—O37—Si1	−132.0 (5)	N2D—N1D—C1D—C2D	173.2 (11)
O4—W1—O37—Si1	−44.3 (5)	N4D—C1D—C2D—N5D	−120.5 (12)
O5—W1—O37—W3	−94.6 (3)	N1D—C1D—C2D—N5D	67.3 (15)
O3—W1—O37—W3	−0.6 (3)	C3D—N5D—C2D—C1D	170.9 (11)
O2—W1—O37—W3	92.8 (3)	C6D—N5D—C2D—C1D	48.3 (14)
O4—W1—O37—W3	−179.4 (3)	C6D—N5D—C3D—C4D	−52.3 (14)
O5—W1—O37—W2	174.0 (3)	C2D—N5D—C3D—C4D	−176.6 (10)
O3—W1—O37—W2	−92.0 (3)	C5D—O1D—C4D—C3D	−60.5 (13)
O2—W1—O37—W2	1.4 (3)	N5D—C3D—C4D—O1D	56.9 (15)
O4—W1—O37—W2	89.2 (3)	C4D—O1D—C5D—C6D	63.1 (12)
O10—W3—O37—Si1	−168.1 (14)	C3D—N5D—C6D—C5D	52.1 (12)
O11—W3—O37—Si1	39.6 (5)	C2D—N5D—C6D—C5D	174.2 (10)
O7—W3—O37—Si1	132.9 (5)	O1D—C5D—C6D—N5D	−59.5 (12)
O12—W3—O37—Si1	−49.9 (5)		

### Hydrogen-bond geometry (Å, °)

**Table:**

*D*—H···*A*	*D*—H	H···*A*	*D*···*A*	*D*—H···*A*
N3A—H3NA···O1W^i^	0.88	2.01	2.831 (16)	155
N3B—H3NB···O2W^ii^	0.88	1.98	2.812 (17)	157
N3C—H3NC···O4W^iii^	0.88	1.83	2.69 (2)	167
N3D—H3ND···O3W	0.88	1.84	2.69 (2)	163
N5A—H5NA···O2W	0.90	1.93	2.797 (19)	162
N5B—H5NB···O1W	0.90	1.90	2.740 (18)	154
N5C—H5NC···N1D^ii^	0.90	1.99	2.89 (2)	172
N5D—H5ND···N1C^i^	0.90	2.07	2.946 (18)	164
O1W—H1WA···O5W^iv^	0.85	1.91	2.691 (13)	153
O1W—H1WB···O19	0.85	1.95	2.779 (12)	167
O2W—H2WA···O6W	0.85	1.80	2.601 (14)	157
O2W—H2WB···O12^v^	0.85	2.10	2.924 (14)	162
O3W—H3WA···O5W^i^	0.85	2.27	2.877 (16)	128
O3W—H3WB···O35^i^	0.85	2.12	2.937 (12)	160
O4W—H4WA···O2^vi^	0.85	2.28	2.862 (14)	126
O4W—H4WB···N4D	0.85	2.50	2.89 (2)	109
O5W—H5WA···N2D^ii^	0.85	2.31	3.155 (18)	175
O5W—H5WB···O26	0.85	2.17	2.944 (14)	151
O5W—H5WB···O28	0.85	2.45	3.118 (14)	136
O6W—H6WA···O17^vi^	0.85	2.17	2.923 (15)	148
O6W—H6WA···O26^v^	0.85	2.28	2.858 (17)	125
O6W—H6WB···N2C^vii^	0.85	2.07	2.911 (17)	172

Symmetry codes: (i) *x*, *y*−1, *z*; (ii) *x*, *y*+1, *z*; (iii) *x*−1, *y*+1, *z*; (iv) *x*, *y*, *z*−1; (v) *x*+1, *y*, *z*; (vi) *x*+1, *y*, *z*+1; (vii) *x*+1, *y*−1, *z*.
